# Pseudo-immortalization of postnatal cochlear progenitor cells yields a scalable cell line capable of transcriptionally regulating mature hair cell genes

**DOI:** 10.1038/srep17792

**Published:** 2015-12-07

**Authors:** Brandon J. Walters, Shiyong Diao, Fei Zheng, Bradley J. Walters, Wanda S. Layman, Jian Zuo

**Affiliations:** 1 Dept. of Developmental Neurobiology, St. Jude Children’s Research Hospital, Memphis, TN 38105, USA

## Abstract

The mammalian cochlea is a highly specialized organ within the inner ear. Sensory hair cells (HC) in the cochlea detect and transduce sound waves into electrical impulses that are sent to the brain. Studies of the molecular pathways regulating HC formation are hindered by the very sparse nature of HCs, where only ~3300 are found within an entire mouse cochlea. Current cell lines mimic certain aspects of HCs but lack terminal HC marker expression. Here we successfully “pseudo-immortalized” cochlear progenitor cells using the “conditional reprogramming” technique. These cells, termed “Conditionally Reprogrammed Otic Stem Cells” (CR-OSC), are able to bypass the senescence inherent to cochlear progenitor cells without genetic alterations, allowing for the generation of over 15 million cells from a single cochlea. These cells can be differentiated and up-regulate both early and terminal differentiation genes associated with HCs, including the terminal HC differentiation marker *prestin*. CR-OSCs also respond to known HC cues, including upregulation of HC genes in response to *Atoh1* overexpression, and upregulation of *prestin* expression after thyroid hormone application. Overall, we describe the creation of a HC line capable of regulated expression of HC genes that can easily be recreated in any laboratory from any mouse of interest.

Auditory hair cells (HCs) are mechanosensory cells in the cochlea that are critical for audition. HCs are highly specialized cells that are present in relatively low abundance with approximately 3300 HCs per mouse cochlea^1^. Two types of HCs exist within the cochlea, the inner hair cells, which are primarily responsible for the detection and transduction of sound into neuronal signaling, and the outer hair cells (OHCs), which are electromotile and act as a cochlear amplifier^2^,^3^,^4^. Electromotility of OHCs is controlled by the non-traditional motor protein prestin^5^, which is coded for by the Slc26a5 gene, and is a unique protein expressed in OHCs. Without the amplification provided by prestin/OHCs, mice suffer a substantial loss of hearing^3^,^4^ demonstrating the importance of this protein for auditory function.

Despite the crucial role for prestin in the cochlea, relatively little is known about the transcriptional regulation of *prestin*. Thyroid hormone (TH) was the first factor discovered to regulate *prestin* expression based on observations that hypothyroidism can result in hearing abnormalities^6^,^7^,^8^. It was later demonstrated that TH binds directly to and activates *prestin*^9^,^10^,^11^. Later studies correlated transcription factors such as Pou4f3 with *prestin* expression^11^, but these studies have been unable to further clarify the mechanisms underlying these correlations. One of the major limiting factors for the study of *prestin* regulation is the lack of an appropriate system to analyze. Most studies to date have been performed in cochlear explants, vastly limiting the material available, the speed at which experiments can be done, and dramatically increasing the cost of the experiment. Indeed, this is true for investigations into the regulation of any genes or proteins expressed specifically in HCs.

To bridge this gap, multiple cell lines have been developed to aid in the study of HC development or to be used as screening tools for the prevention of ototoxicity. Many of these cell lines were created from the “immorto-mouse”^12^,^13^,^14^ and exhibit several aspects of HCs^15^,^16^. These cell lines have been used to identify dozens of compounds, and pathways that ameliorate ototoxic effects of cisplatin or aminoglycoside antibiotic treatment^17^,^18^,^19^. Although these cell lines have proven useful for ototoxic screening studies, they have not been ideal for studying terminal HC differentiation. Additionally, studies have shown that some of these cell lines have begun to show significant phenotypic drift and are no longer sensitive to aminoglycoside induced cell death^20^,^21^.

Lineage restricted auditory progenitor cells, often called otic spheres, or otic stem cells, can be isolated from embryonic and postnatal cochleae^22^,^23^,^24^, and differentiated into cells which bear many hallmarks of a HC^22^,^23^,^24^,^25^, including the ability to express the terminal HC gene, *prestin*, under differentiating conditions^25^. While promising, these cells can only be grown for a few generations, yielding only thousands of cells^25^, limiting their use for large scale studies. In other epithelial tissues, such as breast and prostate, the passage number limitations inherent to lineage restricted progenitor cells can be overcome by “conditional reprogramming” (CR) of the cells^26^,^27^,^28^. This procedure involves growing lineage restricted progenitor cells on a layer of feeder cells, while treating the cells with an inhibitor of rho associated kinases (ROCK). Amazingly, this procedure allowed for the unrestricted growth of the breast and prostate progenitor cells, without compromising their ability to differentiate into mature breast or prostate tissues when put into differentiation conditions^26^. Since the organ of Corti is also an epithelial tissue, we asked whether the CR procedure could be applied to otic progenitor cells, and if the CR-otic progenitor cells could prove useful for the study of terminal hair cell genes, such as *prestin*.

In this study, we exposed otic progenitor cells to CR conditions (termed Conditionally Reprogrammed Otic Stem Cells, CR-OSCs) and found that they respond similarly to CR-breast and prostate tissue. Primarily, CR-OSCs can be grown for many generations and can easily generate 15 million cells from 10 primary otic spheres. Similar to breast and prostate cells, CR-OSCs demonstrated an increase in the expression of telomerase over passage number. Remarkably, once we removed these cells from CR conditions and transferred them into differentiation conditions, the CR-OSCs transcriptionally upregulated nearly every tested gene for hair cell maturation, including the terminal maturation marker *prestin*. Finally, we tested whether CR-OSCs could regulate transcription of hair cell genes in response to two known pro-HC manipulations: ectopic expression of Atoh1, an early pro-HC transcription factor, and application of TH which promotes *prestin* transcription. After ectopic Atoh1 expression, early HC markers were upregulated, mirroring what has been observed in previous studies^29^,^30^,^31^,^32^,^33^,^34^. Consistent with known effects of TH on HCs in cochlear explants^11^, application of TH (either T_3_ or T_4_) to CR-OSCs resulted in a dramatic upregulation of *prestin* expression. Combined, these data demonstrate that CR-OSCs can respond to pro-HC manipulations via the upregulation of HC-specific transcripts.

In total we have described the creation of a novel, easy-to-generate cell line capable of expressing many genes characteristic of differentiated hair cells including the terminal differentiation gene *prestin*. The ease of this procedure allows any laboratory to quickly create CR-OSCs from any mouse model, including genetically modified mouse lines, or crosses of genetically modified mouse lines. Thus, a large number of CR-OSCs can be obtained from Cre/LoxP, rtTA/tTA, or fluorescent reporter mice. These can then be used to expedite fate-mapping and knock-out, and knock-in studies, as well as the development of reporter and mutant hair cell lines to study the regulation of HC specific genes. Additionally, creating cell lines from genetic mouse models in this way should provide for less cell-to-cell variability than viral transduction studies. Though, our work here shows that CR-OSCs can also be virally transduced allowing many ways for the researcher to ask questions about gene function in otic progenitor cells. Finally, CR-OSCs can produce vast quantities of cells making large scale downstream applications feasible; from next-generation sequencing to high throughput screening. CR-OSCs will provide hearing researchers a powerful new tool to investigate the genetic regulation of HC maturation and potentially regeneration.

## Methods

### Animals

The mouse strains used in this study include: prestin-CreER^35^, CAG-Cre^36^, Ai14-tdTomato^36^, and prestin-YFP (Yamashita *et al.*, in press). All animal work conducted during the course of this study was approved by the Institutional Animal Care and Use Committee at St. Jude Children’s Research Hospital and was performed according to NIH guidelines.

### Harvesting Otic Spheres

Whole organs of Corti were separated from the stria vascularis and from the modiolus by micro-dissection from postnatal day (P) 2-4 mice, into Hank’s Balanced Salt Solution with no calcium and no magnesium (Life Technologies), and digested with 0.125% trypsin-EDTA for 10 minutes. Enzymatic digestion was halted with the addition of 10 mg/mL soybean trypsin inhibitor (Life Technologies) and 1 mg/mL DNase I (Sigma Aldrich). Cells were triturated gently 20 times using a 1000 μL pipette tip to be single cell suspension, and passed through a 40-μm cell strainer. The cells were grown in renewal media consisting of: DMEM/F12 (Life Technologies) with supplement of 1× N2 (Life Technologies), 1 × B27 (Life Technologies), 50 ng/mL Ampicillin (Fisher Scientific), mouse epidermal growth factor (20 ng/ml), mouse basic fibroblast growth factor (10 ng/ml), insulin-like growth factor-1 (50 ng/mL; all growth factors purchased from R&D Systems) and heparin sulfate (50 ng/mL; Sigma). After 1–3 days, solid spheres were identified and harvested.

### Conditional Reprogramming of Otic Spheres

Solid otic spheres were placed into a 6-well plate coated in a layer of mitomycin c (Enzo Life Sciences) inactivated (10 μg/mL, 3 hours) 3T3-J2 cells (Swiss 3T3 fibroblasts J2 strain, less than passage 20, 6 × 10^5^/well, gift from Dr. Schlegel^26^), NIH/3T3 cells (ATCC CRL-1658), or primary MEFs in CR media at 5% CO_2_, and 37 °C. CR media consisted of F medium (3:1 (v/v) F-12 Nutrient Mixture: (Ham)–Dulbecco’s modified Eagle’s medium (DMEM, Invitrogen), 5% fetal bovine serum (FBS, Life Technologies), 0.4 μg/mL hydrocortisone (Sigma-Aldrich), 5 μg/mL insulin (Sigma-Aldrich), 8.4 ng/mL cholera toxin (Sigma-Aldrich), 10 ng/mL epidermal growth factor (Invitrogen), and 24 μg/mL adenine (Sigma-Aldrich) with addition of 5 μM Y-27632 (ROCK inhibitor, Enzo Life Sciences)^26^ for 7 days (medium change every 2–3 days) when large mitotically active colonies of CR-OSCs were identified and harvested manually, and placed into differentiation conditions^22^,^25^: poly-L-ornithine, and fibronectin treated 6-well plates, in media containing: DMEM/F12 (Life Technologies) supplemented with 1x N2 (Life Technologies), 1x B27 (Life Tech), 50 ng/ml Ampicillin (Fisher Scientific) for 14 days (1 medium change after 7 days). For passage of CR-OSCs, mitotically active colonies were identified and exposed to TrypLE (Life Technologies) for 15 min at room temperature continuously monitored under a microscope. After 15 min the CR-OSC colonies were preferentially digested and carefully removed from the feeder cells. Any colonies that were not removed by 15 min were manually scraped from the surface, and harvested. CR-OSCs were spun down at 500 g for 10 min, and re-suspended in fresh CR media. 50,000 cells were re-plated onto a 6-well plate coated with feeder cells to start the next passage.

### Cell Culture

Human embryonic kidney cells (HEK) were obtained from ATCC (CRL-1573, less than 20 Passages); NIH/3T3 cells (ATCC CRL-1658) were a generous gift from Dr. Suzanne Baker (St. Jude Children’s Research Hospital); and the primary mouse embryonic fibroblasts (MEFs) were gifted by Dr. Guillermo Oliver (St. Jude Children’s Research Hospital). These cells were maintained at 37 °C with 5% CO_2_ in DMEM (Life Technologies) supplemented with 10% FBS (Life Technologies) and 1x penicillin/streptomycin (Life Technologies). HEI-OC1 cell line (A gift from Dr. Kalenic and the House Research Institute^14^) was grown under permissive conditions: 10% CO2, 33 °C, and DMEM + 10% FBS + 50 ng/mL ampicillin (Life Technologies).

### Viral transduction

Adeno-Associated Virus (AAV) subtypes 2/5 overexpressing *Atoh1* or *GFP* (Vector Bioloabs) were added to a final concentration of 2.5 × 10^11^ genome copies/mL into a 96-well plate containing 1–2 large CR-OSC colonies (approximately 5,000 cells) or 5,000–10,000 HEK or 5,000 HEI-OC1 cells for either 2 or 7 days after which the mRNA was harvested and analyzed.

### Quantitative Real Time PCR

Total RNA was harvested using RNA-Stat 60 (Tel-Test Inc.), and 200 ng of total RNA was converted to cDNA using High-Capacity cDNA Reverse Transcription Kit (Life Technologies), then diluted to 1 ng/μL cDNA in ddH_2_0. 2 ng were used for multiplexed qPCR using Taqman Mastermix (Life Technologies) following the manufacturer’s instructions. qPCR was performed using a Mastercycler Realplex^2^ (Eppendorf) real time PCR machine.

### qPCR Primers

Primer/probes were obtained from Life Technologies FAM: Atoh1 (Mm00476035_s1), Pou4f3 (Mm04213795_s1), myosin VI (Mm00500651_m1), myosin VIIa (Mm01274015_m1), parvalbumin (Mm00443100_m1), otoferlin (Mm00453306_m1), prestin (Mm00446145_m1), VGlut3 (Mm00805413_m1), telomerase (Mm00484957_m1). VIC: 18 s (4319413E).

### Immunohistochemistry

Differentiated CR-OSCs were fixed with 4% paraformaldehyde in phosphate-buffered saline (PBS) at room temperature for 15 minutes. Immunostaining was performed with the myosin VI rabbit polyclonal antibody (1:200, Proteus BioSciences) followed by Tyramide Signal Amplification (TSA) kit (Life Tech) following the manufacturer’s instructions, or with GFP chicken polyclonal antibody (1:500, Abcam) followed by fluorescein-conjugated secondary antibody. Nuclei were visualized by a 20 minute incubation with Hoechst33342 (1:2000, Life Tech) in PBS at room temperature. Cells were imaged using an Operetta High Content Imaging System (Perkin Elmer) or a Zeiss LSM 780 inverted confocal microscope (Zeiss).

### Statistics

Statistics were performed using Origin 8.5 (OriginLab). Samples were compared using a standard two sample T test, and where multiple comparisons were performed the p-values were adjusted using the Bonferroni correction.

## Results

### Lineage Restricted Otic Progenitor Cells can be Pseudo-immortalized

**Figure 1:**
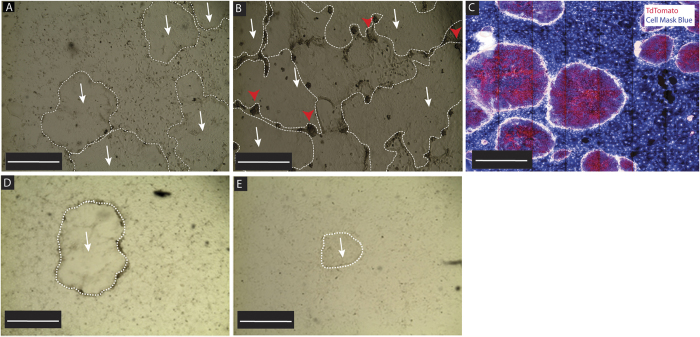
Lineage Restricted Otic-Stem cells can be conditionally reprogrammed. (**A**) P2-P4 otic-spheres were placed into conditional reprogramming conditions and grown for 7 days. Large growing colonies (white arrows and circled by white dashed lines) were observed in the mitotically inactive feeder cells. (**B**) After 14 days feeder cells are actively displaced from the plate (red arrow heads) as the growing colonies overtake the plate. (**C**) Otic-spheres were harvested from Rosa26-CAG-TdTomate mice and placed into conditional reprogramming conditions for 7 days, after which they were stained with cell mask blue and imaged. Large TdTomato positive colonies were observed growing in the feeder cells when examined under fluorescent microscopy. (**D**,**E**) Otic-spheres grew on NIH3T3 cells (**D**) or primary MEFs (**E**) for 14 days.

To test if CR conditions developed for breast/prostate spheres could be successfully applied to the organ of Corti, we isolated otic-spheres from postnatal day P2-4 wild-type (wt) mouse cochleae and exposed them to the CR conditions. After 7 days, large colonies were observed growing in the mitotically inactive feeder cells (Fig. 1A, white arrows, colonies circled by white dashed lines) and eventually displaced the feeder cells (Fig. 1B, dead feeder cells indicated by red arrow heads). To ensure these colonies were from our mice and not feeder cell contamination, we performed the same procedure using *Rosa26-CAG-TdTomato* mice^36^, where all cells within the mice express the red fluorescent protein tdTomato. After 7 days, large, red colonies were seen growing in, and actively displacing the feeder cells which did not exhibit any red fluorescence (Fig. 1C). To test the requirement of Y-27632 or the feeder cells, we attempted to reprogram otic spheres without either the Y-27632 or without the feeder cells and observed no reprogramming in the absence of either of these factors, indicating that both ROCK inhibition and the presence of the feeder cells are necessary for the process (data not shown).

To extend the general applicability, we used MitoC-treated NIH3T3 cells or primary MEFs as the feeders. Interestingly, we found that although otic-spheres could grow on both NIH3T3 and MEFs, the growing speed was slower (Fig. 1D,E), and no colonies were observed after second passage. These results suggest J2 cells are the most suitable CR feeder cells.

### Characterization of Conditionally Reprogrammed Otic Stem Cells (CR-OSCs)

**Figure 2:**
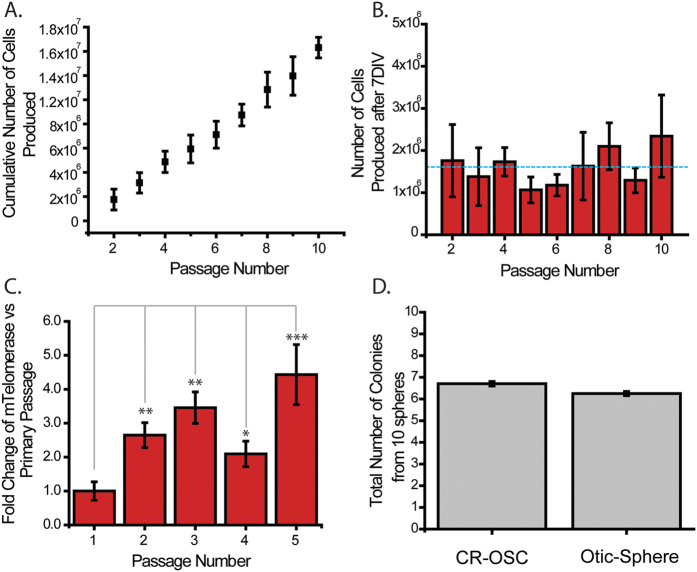
Characterization of Conditionally Reprogrammed Otic Stem Cells (CR-OSCs). (**A**) 50,000 CR-OSC cells were plated starting at passage 2 and grown for 7 days. After 7 days the cells were differentially trypsinized from the remaining feeder cells, counted, and 50,000 of them were re-plated for the next passage. The cumulative total of cells produced during this period was plotted against passage number (n = 3, slope = 1.534 × 10^6^, R^2^ = 0.992). (**B**) The number of cells produced after 7 days in culture was plotted against passage number (n = 3). Dotted blue line represents the overall average across all generations, 1.61 × 10^6^. (**C**) The relative expression of telomerase normalized to the 18s ribosomal subunit was compared for the first 5 passages of CR-OSCs against passage 1 (n = 10). (**D**) Otic-spheres were isolated and trypsinized into a single cell suspension. Half of the suspension was plated into CR conditions, and the other half plated to form new secondary spheres. After 7 days the number of new CR colonies or secondary spheres were counted and plotted (n = 15). *indicates p < 0.05 corrected by Bonferroni method. Mean ± S.E.M.

To better characterize the CR-OSC cells, we initially monitored their growth over 10 generations and asked how their growth rate changed with each passage. CR-OSCs were maintained in 1 well of a 6-well plate, and continually passaged for 10 passages. Every 7 days CR-OSCs were harvested, counted, and 50,000 CR-OSCs were re-plated for the next passage. CR-OSCs generated over 15 million cells throughout the 10 passages studied (Fig. 2A), a vast improvement to the tens of thousands of cells which can be produced from otic spheres prior to the onset of senescence^25^. Furthermore, the growth rate did not appear to change over the 10 passages (Fig. 2B); averaging approximately 1.6 million cells generated every passage (Fig. 2B, dashed line). Previous work done on breast and prostate spheres demonstrated a robust increase in telomerase expression during the CR passages. Similarly, CR-OSCs demonstrated an increase in the transcription of telomerase for several passages (Fig. 2C, 1° (primary passage)-2° (secondary passage) p = 0.005, 1°–3° p = 0.003, 1°–4° p = 0.028 1°–5° p = 7 × 10^−4^). Finally, we sought to differentiate between two different hypotheses that could explain the increased proliferation observed in the CR-OSCs. It is possible that the prolonged proliferation seen in CR-OSCs was due to the CR conditions recruiting normally non-dividing cells to proliferate, or alternatively the existing pool of dividing cells could have been coaxed into continued proliferation. To discriminate between the two, we trypsinized primary otic spheres into a single cell suspension, and plated half of the suspension into CR conditions, and half to form new secondary otic spheres. We reasoned if equal numbers of new secondary spheres formed as did CR-OSC colonies it would eliminate the possibility that normally non-dividing cells were recruited when the cells are in CR conditions. After 7 days we counted the new secondary spheres and compared it against CR-OSC colonies. We observed no significant difference between the two (Fig. 2D, n = 15, p = 0.75), implying that normally non-dividing cells are not the source of the increased proliferation seen in CR-OSCs.

### CR-OSCs Transcriptionally Up-regulate Hair Cell Genes in Response to Differentiation Conditions

**Figure 3:**
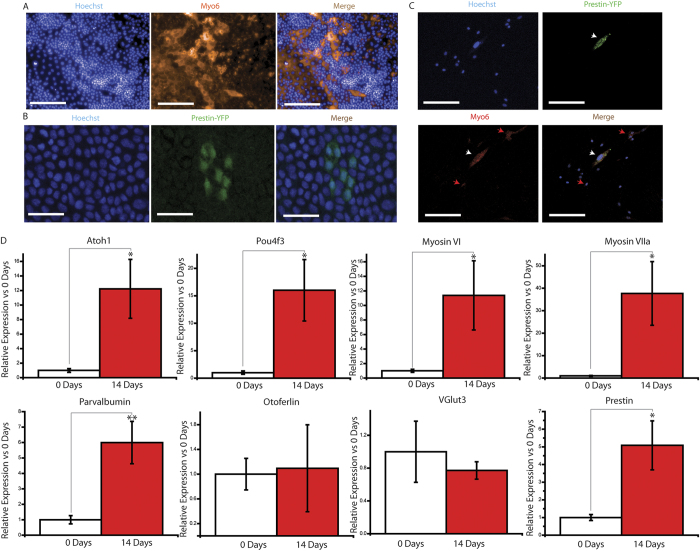
CR-OSCs Transcriptionally Upregulate Hair Cell-specific Genes in Response to Differentiation Conditions. (**A**) Primary passage CR-OSC colonies were manually harvested and plated into differentiation conditions. After 14 days the cells were fixed, stained for myosin VI (red) or Hoechst (blue), and imaged. (**B**) Primary passage CR-OSC colonies from Prestin-YFP mice were manually harvested and plated into differentiation conditions for 21 days after which the cells were fixed and immune-labeled by YFP antibody. (**C**) Primary passage CR-OSCs were differentiated for 21 days and stained for myosin VI (red), YFP (green) and Hoechst (blue). (**D**) Primary passage CR-OCS colonies were harvested before (0 Days) and after (14 Days) differentiation. The expression of transcripts were normalized to 18s RNA. N = 15 for all conditions. *indicates p < 0.05. Mean ± S.E.M.

To characterize how CR-OSCs regulate known HC genes in response to a differentiation cue, we manually harvested and plated primary CR-OSC colonies into differentiating conditions. After 14 days we performed immunohistochemistry against a common, highly expressed hair cell marker, myosin VI, and observed 5.97 ± 0.96% myosin VI positive cells in differentiated CR-OSCs (Fig. 3A). To examine if CR-OSCs also express prestin, a terminal outer hair cell differentiation marker, we cultured otic spheres from prestin-YFP mice, in which the endogenous *prestin* is tagged with YFP. Interestingly, we found less than 1% CR-OSCs expressed YFP signal after 21 days (Fig. 3B). Double labeling myosin VI and prestin-YFP showed all YFP positive cells are myosin VI positive (Fig. 3C, white arrow heads), indicating that a portion of myosin VI-positive cells terminally differentiated and expressed prestin.

To better characterize the regulation of HC markers, we performed quantitative real time PCR (qPCR) against a panel of eight HC markers spanning early to terminal maturation states (Atoh1, Pou4f3, myosin VI, myosin VIIa, parvalbumin, otoferlin, VGlut3, and prestin) and compared expression of these markers between 0 and 14 days of differentiation conditions. Under both situations all eight genes were expressed at detectable levels (Fig. 3D), but Atoh1 (p = 0.014), Pou4f3 (p = 0.019), myosin VI (Fig. 3F, p = 0.046), myosin VIIa (p = 0.025), parvalbumin (p = 0.006), and prestin (p = 0.013) were significantly upregulated under differentiating conditions for 14 days.

### CR-OSCs Transcriptionally Upregulate Hair Cell Genes in Response to Differentiation Condition Regardless of Passage Number

**Figure 4:**
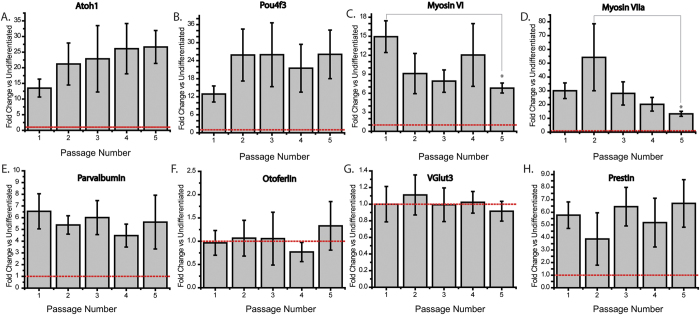
The Effect of Passage Number on the Ability of CR-OSCs to Transcriptionally Upregulate Hair Cell Genes in Response to Differentiation Conditions. (**A–H**) CR-OSC colonies were continually passaged for 5 generations and CR-OSCs were manually harvested after each passage and plated into differentiation conditions for 14 days, after which the colonies were harvested and the relative expression (normalized to 18s) was calculated for: Atoh1 (**A**), Pou4f3 (**B**), myosin VI (**C**) myosin VIIa **(D**) parvalbumin (**E**), otoferlin (**F**), VGlut3 (**G**), or prestin (**H**) and plotted relative to expression levels of the primary passage. N = 15 for all conditions. *indicates p < 0.05 corrected by Bonferroni. Mean ± S.E.M.

We have demonstrated that CR-OSCs can be passaged repeatedly. Here, we sought to characterize the expression levels of HC genes against the passage number. We passaged CR-OSCs through 5 passages, manually harvested and differentiated CR-OSC colonies after each passage, followed by qPCR. The first 4 passages had no change in the expression levels of any tested gene (Fig. 4A–H). However, after the fifth passage, *myosin VI* (Fig. 4C, p = 0.029) and *myosin VIIa* (Fig. 4D, p = 0.013) were expressed at levels significantly lower than their maxima (passage 1 for myosin VI, and passage 2 for myosin VIIa), though still significantly higher than undifferentiated CR-OSCs (red dashed line, *myosin VI* 6.75 fold increase over undifferentiated, *myosin VIIa* 14.5 fold increase over undifferentiated). All other HC genes (*Atoh1*, *Pou4f3*, *Parvalbumin*, *otoferlin*, *prestin*, *VGlut3*) demonstrated no change in expression levels after any passage assayed by quantitative real time PCR.

### CR-OSCs Upregulate *Pou4f3*, *Myosin VI*, and *Otoferlin* in Response to *Atoh1* Overexpression

**Figure 5:**
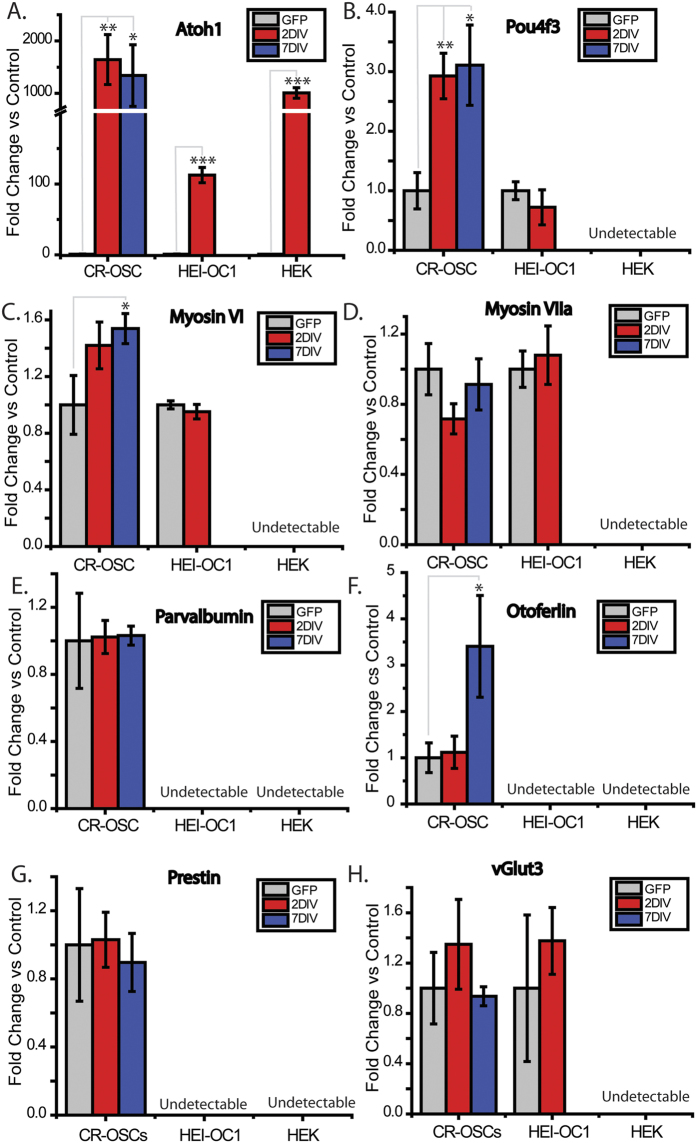
CR-OSCs Upregulate Pou4f3, Myosin VI, and Otoferlin in Response to Atoh1 Overexpression. (**A–H**) Primary CR-OSC colonies were manually harvested and plated into differentiation conditions for 14 days. After 7, or 12 days (corresponding to 7 days or 2 days of viral transduction) 1.0 × 10^12^ genome copies/mL of AAV-Atoh1 or AAV-GFP virus was added to the media. Similarly HEK or HEI-OC1 cells were plated and 1.0 × 10^12^ genome copies/mL of virus was added for 2 days. Following treatment, cells were harvested and expression of Atoh1 (**A**), Pou4f3 (**B**), myosin VI (**C**) myosin VIIa (**D**) parvalbumin (**E**), otoferlin (**F**), VGlut3 (**G**), or prestin (**h**) was determined, normalized to 18s. Data is plotted against the control GFP viral transductions. N = 10 for all conditions. *indicates p < 0.05 corrected by Bonferroni method. Mean ± S.E.M.

We next tested if CR-OSCs responded to a common pro-HC cue, overexpression of *Atoh1*, which has been demonstrated to upregulate HC-specific genes in non-HCs and promote an immature HC fate *in vivo*^29^,^31^,^37^. We transduced AAV containing CAG driven *Atoh1* into CR-OSCs, as well as an AAV containing a CAG driven GFP as a negative control. We also transduced HEK cells and HEI-OC1 cells, a hair cell line commonly used in testing small molecule compounds for otoprotection, to compare how different cell lines performed in response to *Atoh1* overexpression. We chose to use the permissive conditions for the HEI-OC1 cells, as these conditions are the mostly commonly used for screening, express several HC markers^14^, and have a substantially lower rate of cell death^14^. AAV-*Atoh1* was transduced during differentiation of CR-OSCs for either 2 days or 7 days, or for only 2 days in HEK and HEI-OC1 cells due to their proliferative nature. After transduction with AAV-*Atoh1*, colonies were harvested and assayed by qPCR. *Atoh1* levels were significantly higher in all cases (Fig. 5A, CR-OSC 2DIV p = 0.009; CR-OSC 7DIV p = 0.05; HEK 2DIV p = 2.9 × 10^−4^; HEI-OC1 p = 6.76 × 10^−6^) but only CR-OSCs had upregulation of HC genes in response to *Atoh1* overexpression (Fig. 5B: *Pou4f3* 2DIV, p = 0.009; *Pou4f3*, 7DIV p = 0.024; Fig. 5C *myosin VI*, 7DIV p = 0.035; Fig. 5C *otoferlin*, 7DIV, p = 0.031). Notably, overexpressing Atoh1 failed to induce *prestin*, consistent with previous studies that demonstrated that overexpression of Atoh1 was not sufficient to force terminal maturation of HCs^29^,^31^,^37^. Thus, our results indicate CR-OSCs respond to Atoh1 overexpression similar to otic progenitor cells under *in-vivo* or *ex-vivo* conditions, while HEK and HEI-OC1 cells have almost no response.

### CR-OSCs Upregulate Prestin in Response to Thyroid Hormone Treatment

**Figure 6:**
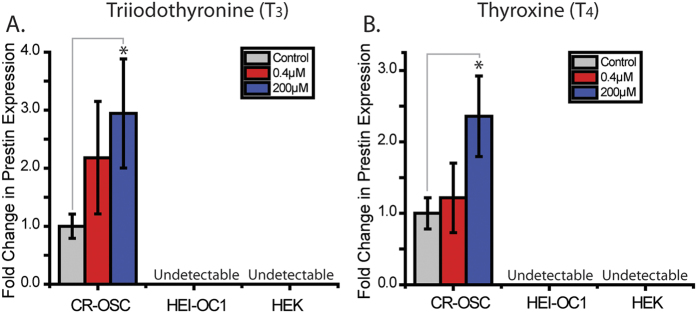
CR-OSCs Upregulate Prestin in Response to Thyroid Hormone Treatment. (**A**,**B**) Primary CR-OSC colonies were manually harvested and placed into differentiation conditions for 14 days. HEK cells or HEI-OC1 cells were also tested. Triiodothyronine (**A**) or Thyroxine (**B**) were added to the culture media for 2 days at either 400 nM (red) or 200 μM (blue). N = 10 for all conditions. *indicates p < 0.05 corrected by Bonferroni method. Mean ± S.E.M.

Finally, we asked whether CR-OSCs can up-regulate *prestin* expression in response to known mediators of *prestin* mRNA expression. We tested the responsiveness of CR-OSCs to the prohormone Thyroxin (T_4_) and the 4-fold more potent form, Triiodothyrone (T_3_), which has been described to potentiate prestin expression in OHCs^11^. Quantitative PCR revealed that application of either T_3_ (Fig. 6A, p = 0.032) or T_4_ (Fig. 6B, p = 0.042) caused a significant upregulation of prestin mRNA in CR-OSCs, while no detectable level of prestin was observed in either HEK or HEI-OC1 cells following similar treatments. These results again demonstrate the similarities between the CR-OSCs and cochlear OHCs in the upregulation of *prestin* expression by thyroid hormones.

## Discussion

In this study we explored the conditional reprogramming technique, which was designed to allow the unlimited proliferation of breast and prostate progenitor cells without affecting their lineage restricted differentiation potential^26^. Since breast and prostate cells as well as the cells that comprise the organ of Corti are all epithelial, we hypothesized that the conditional reprogramming technique would allow for the continued proliferation of otic progenitor cells. We found that the conditional reprogramming technique was able to increase the number of cells generated from otic progenitor cells from the 10^4^ cells currently reported^25^ to 10^7^ CR-OSCs. Additionally, we did not observe any senescence before the experiments were arbitrarily ended at 10 passages. We were even able to passage one line for 25 passages. These data suggest that if one desired greater numbers of CR-OSCs, the procedure could easily be scaled up by seeding with more than 50,000 cells, or by continuing to plate cells beyond the 10^th^ passage. Additionally, CR-OSCs appear to recapitulate the ability of CR treated breast and prostate progenitor cells to differentiate into their lineage-restricted cell types when placed into differentiation conditions. CR-OSCs actively up-regulated numerous HC genes including early differentiation genes like *Atoh1* and *Pou4f3* as well as terminal differentiation genes such as *prestin* in response to differentiation conditions.

CR-OSCs offer many advantages over traditional methodologies: first and foremost, the ability to generate millions of cells from a single cochlea; cells that allow for monitoring and manipulation of the expression of many HC specific genes which should allow researchers to save time, resources, and animals when asking questions pertaining to transcriptional regulation of HC-specific genes. Second, the simplicity of this procedure allows for any laboratory to generate CR-OSCs. The conditional reprogramming procedure involves only 2 steps: 1) plating otic progenitors onto a layer of mitotically inactivated 3T3 feeder cells; 2) addition of a Rho associated kinase (ROCK) antagonist to the F-media. Third, this procedure is transient, and does not involve the manipulation of a cell’s genome to achieve the increased growth. The transient nature and ease of the procedure allows CR-OSC to be generated from a primary culture of otic progenitor cells, and allows one to easily establish and re-establish CR-OSC cell lines from live mice to minimize genetic drift.

Surprisingly, our stock of HEI-OC1 cells completely lacked *prestin* expression even though HEI-OC1 cells were previously shown to express prestin by immunohistochemistry in both the permissive conditions (the conditions used in this study) and non-permissive conditions^14^. This observation was also evident in the Thyroid hormone and AAV-Atoh1 sets of experiments. The simplest explanation for this observation is that genetic drift has caused HEI-OC1 cells to lose *prestin* expression over the numerous generations that have passed since their development. In fact, drift has previously been reported for some HEI-OC1 populations in regard to aminoglycoside sensitivity^20^,^21^. While CR-OSCs are not exempt from genetic drift, e.g. after 5 generations of progressively passaging CR-OSCs, there was a small but significant reduction in the expression levels of *myosin VIIa* and *myosin VI*, this genetic drift can be minimized by generating CR-OSCs on demand from live mice and keeping the passage numbers below 10 Our observations therefore not only highlight the impact that genetic drift can have on various cell lines, and the need to constantly verify the integrity of the cell line being utilized, but suggest an advantage of the CR-OSC technique in the ability of every investigator to produce these cells in their own laboratory, and not rely on cell line banks or other investigators for their cells.

Another advantage of the CR-OSC method that is of great importance is that CR-OSCs can be derived from *any* mouse model available, opening up vast resources of genetically modified mouse strains to use in conjunction with CR-OSCs. Thus, cell lines with fluorescent or luminescent reporters can be easily derived for reporter expression analysis in response to pharmacologic or genetic manipulation. Similarly, experiments involving genetic mutations, knock-outs, or overexpression can be tested on large numbers of cochlear cells with relative ease. This is particularly useful in cases where crosses of mice carrying multiple transgenic alleles yield small numbers of double, triple, or quadruple positive mutants, or in cases where the desired genetic manipulations are lethal at embryonic or perinatal ages. Furthermore, the large numbers of cells derived from each cochlea provides enhanced investigative capability in probing the molecular mechanisms or consequences of genetic manipulations. For example fluorescence activated cell sorting (FACS), and whole genome (DNA-seq or ChIP-seq), whole transcriptome (RNA-seq), or whole proteome (mass spectrometry) experiments are less costly and more expedient when large numbers of cells are available for use. In this way, investigators can garner large datasets resulting from a given genetic manipulation or from the enrichment of a cell population that expresses a fluorescent reporter without having to breed, euthanize, and dissect and pool cochleae from large numbers of mice simultaneously.

Similarly, CR-OSCs are readily amenable for use in high throughput screening strategies. Even after being passaged to generated sufficiently large numbers of cells, CR-OSCs retain the ability to respond to manipulations that are known to promote a HC fate (*Atoh1* overexpression^29^,^30^,^31^,^32^), or regulate *prestin* expression (Thyroid hormone^9^,^10^,^11^). CR-OSCs responded to both treatments as expected, upregulating early HC markers in response to *Atoh1* overexpression, and upregulating *prestin* expression in response to thyroid hormone treatment. These results together indicate that CR-OSCs could readily be purposed toward high throughput screens for small molecules or genetic factors that promote the expression of HC-specific genes, including *prestin*, and that ectopic *Atoh1* expression or the application of thyroid hormones could be used as positive control conditions for assay development.

CR-OSCs could potentially be utilized for questions regarding IHC vs OHC fate, as differentiation conditions upregulated the OHC-specific gene *prestin*^4^,^5^,^38^ while having no effect on the IHC-enriched genes *otoferlin*^39^,^40^, and *VGlut3*^41^,^42^. Alternatively, *VGlut3* and *otoferlin* are both synaptic proteins, and may not be upregulated because of the lack of synaptic connections in our conditions. If this scenario is correct, one could hypothesize that co-culturing CR-OSCs with neurons during differentiation may result in the up-regulation of these genes, a hypothesis that would require further testing in future experiments.

Similarly, our AAV transduction experiments provided some unexpected insights. While it is well known that *in vivo Atoh1* transduction can promote immature HC phenotypes, the up-regulation of *otoferlin* was unexpected. These data suggest that the gene networks modulated by *Atoh1* overexpression may be involved in signaling pathways essential for cell fate decisions to produce IHCs, which is consistent with ideas put forth by Jahan *et al.*^43^. Reports have also shown that *otoferlin* is highly expressed in vestibular hair cells^44^, and that *Atoh1* overexpression may select for vestibular type hair cells when overexpressed in the cochlea^45^. Thus, our data may also be supportive of the idea that *Atoh1* overexpression may selectively up-regulate gene targets that are enriched in both vestibular and inner hair cells. Consistent with this notion, AAV mediated transduction of *Atoh1* in CR-OSCs did not result in up-regulation of the OHC-specific gene *prestin*. We were also struck by the lack of response observed in the HEI-OC1 cells following *Atoh1* overexpression. This observation may be due to the lower transduction efficiency displayed by the HEI-OC1 cells (100 fold increase vs. 1000 fold increase in CR-OSCs), but this may also portend the inflexibility of the transcriptional networks inherent to the HEI-OC1 cells. It is also possible that the permissive (proliferative) conditions used in our studies did not allow for the proper up-regulation of normal transcriptional networks. However, the original characterization of the HEI-OC1 cells reported that HEI-OC1 cells express all assayed HC markers in both permissive and non-permissive conditions^14^.

Finally, CR-OSCs may offer a new method for studying otic progenitor cells, specifically with regard to why these cells lose their cellular plasticity during early postnatal development. These data lend evidence that it may be possible to dissect out the factors that lead to the pseudo-immortalization caused by the CR conditions. Indeed, the results presented here suggest that the activities of telomerase and Rho-associated coiled-coil containing protein kinases are likely involved in the senescence of the otic stem cell population. Though, further testing will be required to see if these factors exert similar influence *in vivo*, as well as to determine the necessary factors that are being provided by the feeder cells. Once these factors are better elucidated, researchers may be able to manipulate conditions *in vivo* to help maintain the cellular plasticity that is normally lost in cochlea during postnatal development.

Although CR-OSCs have important implications for understanding hair cell development and the transcriptional regulation of several HC-specific gene products, further characterization of HC bundle formation, electrophysiological responses, and the translation of HC-specific genes into proteins will be required to determine the extent to which these cells have the potential to give rise to functional HCs. Importantly, only about five percent of the CR-OSCs expressed Myo6, and in a portion of this cell population, we observed Prestin expression. On one hand, it reflects the heterogeneity of CR-OSCs similar to many other stem/progenitor cell lines (e.g. HEI-OCs). On the other hand, the nature of the remaining cells remains elusive. A reasonable hypothesis is that they are supporting cell-like because more than 80% of differentiated otic spheres cultured without CR expressed the supporting cell markers pan-Cytokeratin^22^ and Sox2^46^. Here, we attempted to immunostain the differentiated CR-OSCs with antibodies recognizing the supporting cell markers Sox2 and Prox1, as well as the neuronal markers Tuj1 and NeuN, and the mesenchymal cell marker Axin2. However, we detected no convincing signal for these proteins. One possibility is that the immunocytochemistry technique as applied here was not sensitive enough to detect these proteins, although all of the antibodies used did produce positive signal *in vivo*. The other possibility is that the CR condition inhibited the differentiation from otic progenitor cells to supporting cells, or promoted de-differentiation away from the otic lineage, perhaps even to more stem-like cells. Given the interesting and elusive nature of these cells, our future studies are aimed at characterizing this cell population.

In summary, we have described the first otic progenitor cell line capable of producing large quantities of cells that can transcriptionally regulate both early and terminal HC genes and retain the ability to respond normally to cues designed to promote HC fate. However, one of the most valuable aspects of the CR-OSCs is the ability to generate them in any laboratory as needed, and to utilize any genetically modified mouse model. This study provides inner ear researchers a completely novel *in vitro* tool for the study of hair cell development and/or responses to pharmacologic or genetic manipulation and offers the field a primary otic progenitor cell line for high throughput screening strategies aimed at ways to promote the expression of HC-specific genes and possibly even terminal hair cell differentiation.

## Additional Information

**How to cite this article**: Walters, B. J. *et al.* Pseudo-immortalization of postnatal cochlear progenitor cells yields a scalable cell line capable of transcriptionally regulating mature hair cell genes. *Sci. Rep.* **5**, 17792; doi: 10.1038/srep17792 (2015).
