# Corrigendum: Pseudo-immortalization of postnatal cochlear progenitor cells yields a scalable cell line capable of transcriptionally regulating mature hair cell genes

**DOI:** 10.1038/srep21708

**Published:** 2016-02-25

**Authors:** Brandon J. Walters, Shiyong Diao, Fei Zheng, Bradley J. Walters, Wanda S. Layman, Jian Zuo

Scientific Reports 5: Article number: 1779210.1038/srep17792; published online: 12072015; updated: 02252016

The Acknowledgements section in this Article is incomplete.

“We thank Dr. Richard Schlegel for providing the 3T3-J2 cells, Dr. Kalenic and the House Research Institute for providing the HEI-OC1 cells, Dr. Guillermo Oliver for the MEFs, Dr. Suzanne Baker for the NIH 3T3 cells, and Drs. Stefan Heller and Kazuo Oshima for help on isolation of otic-spheres, and Dr. Yannan Ouyang for help on confocal microscope imaging.”

should read:

“We thank Dr. Richard Schlegel for providing the 3T3-J2 cells, Dr. Kalenic and the House Research Institute for providing the HEI-OC1 cells, Dr. Guillermo Oliver for the MEFs, Dr. Suzanne Baker for the NIH 3T3 cells, and Drs. Stefan Heller and Kazuo Oshima for help on isolation of otic-spheres, and Dr. Yannan Ouyang for help on confocal microscope imaging.

This research was supported by funding from the National Institutes of Health grants (R01DC006471 (J.Z.), R21DC013879 (J.Z.), F32DC013832 (W.L), P30CA21765 (St. Jude)), American Lebanese Syrian Associated Charities (ALSAC) of St. Jude Children’s Research Hospital, the Office of Naval Research grants (N000140911014, N000141210191, N000141210775 (J. Z.)), the Hearing Health Foundation (Emerging Research Grants, Brandon J. W. and Bradley J. W.), the National Organization for Hearing Research (NOHR) Foundation (Bradley J. W.), and The Hartwell Foundation (Individual Biomedical Research Award, J. Z.).”

